# Replacement of E-cadherin by N-cadherin in the mammary gland leads to fibrocystic changes and tumor formation

**DOI:** 10.1186/bcr3046

**Published:** 2011-10-26

**Authors:** Ahmed M Kotb, Andreas Hierholzer, Rolf Kemler

**Affiliations:** 1 Department of Molecular Embryology, Max Planck Institute of Immunobiology and Epigenetics, Stuebeweg 51, D-79108 Freiburg, Germany

## Abstract

**Introduction:**

E-cadherin (E-cad; cadherin 1) and N-cadherin (N-cad; cadherin 2) are the most prominent members of the cadherin family of cell adhesion molecules. Although they share many structural and functional features, they are expressed in an almost mutually exclusive manner *in vivo*.

**Methods:**

To explore functional differences between the two cadherins *in vivo*, we recently generated a knock-in line in which N-cad is expressed from the E-cad locus. In combination with a conditional gene inactivation approach, we expressed N-cad in the absence of E-cad (referred to as Ncadk.i.) in alveolar epithelial cells of the mammary gland starting in late pregnancy.

**Results:**

We found that the sole presence of N-cad induces constitutively active fibroblast growth factor (Fgf) signaling and a precocious involution resulting in massive apoptosis of alveolar cells. To block apoptosis, we conditionally deleted one allele of p53 in Ncadk.i. mice and observed a temporal rescue of alveolar morphology and function. However, an accumulation of fibrotic tissue and cysts with increasing age and lactation cycles was observed. This phenotype closely resembled fibrocystic mastopathy (FM), a common disorder in humans, which is thought to precede breast cancer. Concordantly, 55% of Ncadk.i. mice harboring a heterozygous p53 deletion developed malignant and invasive tumors.

**Conclusions:**

Our results demonstrate a possible role for N-cad in the formation of fibrosis and cysts in the mammary gland. Moreover, we show that these lesions precede the development of malignant tumors. Thus, we provide a new mouse model to investigate the molecular mechanisms of fibrocystic mastopathy and the transition from benign to malignant tumors.

## Introduction

Classical cadherins are cell adhesion molecules, which play fundamental roles in the development of multicellular organisms [1]. E-cadherin (E-cad, Cdh1) is the founding member of the cadherin gene family and is essential for the integrity of epithelial tissues. The first epithelial cell layer in the development of mouse embryos, the trophectoderm of blastocysts, requires E-cad for proper function. In later embryonic stages and in adult organisms, E-cad also provides adhesive strength to form polarized epithelial cell layers [2]. N-cadherin (N-cad, Cdh2) is initially expressed at the gastrulation stage when epiblast cells downregulate E-cad and undergo an epithelial-mesenchymal transition (EMT). This process includes the upregulation of N-cad in the nascent mesoderm. In later stages and in adults, N-cad is restricted to neural tissue and cells of mesenchymal origin [3]. Thus, *in vivo*, both cadherins are often expressed in a mutually exclusive manner. E-cad and N-cad share many structural and functional features. Both establish calcium-dependent homophilic cell-cell adhesion with their extracellular domains and are connected with catenins at their intracellular domains [4]. E-cad and N-cad have been shown to be able to interact with the receptors of different growth factors, which suggests that cadherins are involved in the modulation of signaling pathways. For example, E-cad associates with receptors of epidermal growth factors (EGFR) [5], whereas N-cad interacts with receptors of fibroblast growth factors (FGFR) and thus modulates signaling function [6]. We recently addressed the question of interchangeability of the two cadherins by generating a mouse model in which N-cad cDNA was introduced into the E-cad locus. In these mice, N-cad was efficiently expressed in the E-cad expression domains. Homozygous N-cadk.i. mutants fail to form a proper trophectoderm in blastocysts, demonstrating that N-cad cannot replace E-cad in early embryos [7]. However, in a conditional gene replacement approach, we presented evidence that N-cad can support epithelial integrity in the embryonic intestine, but these mice showed a pathologic phenotype after birth [8]. Here, we investigated the capacity of N-cad in supporting epithelial integrity in the mammary gland of adult mice. The mammary gland (MG) consists of lobulo-alveolar structures formed by epithelial cells expressing E-cad [9]. Myoepithelial cells surround the alveoli and provide mechanical stimuli to the epithelial cells of the alveoli during the lactation process. Both alveoli and myoepithelial cells are maintained by signals derived from the stromal compartment [10]. To replace E-cad with N-cad in the mouse mammary gland epithelium, we applied the Ncadk.i. allele together with a conditional E-cad allele [11]. The floxed E-cad allele was ablated using WAP::Cre mice, which express Cre recombinase in the alveolar epithelium of the mammary gland from late pregnancy on and during lactation [12]. We found that the expression of N-cad in the absence of E-cad induces precocious involution and ultimately p53-mediated apoptosis in the alveoli. By conditional ablation of one p53 allele, we could block apoptosis in the alveoli and found that the tissue integrity and function of the alveoli was completely rescued. However, in aged mice and after numerous lactation cycles, we noticed increased fibrosis accompanied by cyst formation, which ultimately led to a loss of lactation capacity. Finally, we observed a transition from these benign lesions to malignant and highly invasive tumors.

## Materials and methods

### Mouse lines and genotyping

The generation of WAP::Cre [12], *Ecad^Ncad/+ ^*[7], *Ecad^fl/fl ^*[11] and *p53^fl/fl ^*[13] mouse lines has been described elsewhere. Heterozygous WAP:Cre;*Ecad^Ncad/+ ^*mice were crossed with *Ecad^fl/fl ^*mice to get WAP::Cre;*Ecad^Ncad/fl ^*mice. Subsequently, WAP::Cre;*Ecad^Ncad/fl ^*and WAP::Cre;*Ecad^fl/fl ^*lines were mated with *p53^fl/fl ^*animals to obtain mice with a WAP::Cre;*Ecad^Ncad/fl^*;*p53^fl/+ ^*and WAP::Cre;*Ecad^fl/fl^*;*p53^fl/+ ^*genotype, respectively. Experimentation with mice was performed according to §4 German Animal Welfare Law and after review by the Institutional Animal Care and Use Committee (IACUC), internal license #KE-iTO-6.

Genotyping was performed by PCR with genomic DNA isolated from mouse tail biopsies using primers for the *Ncad k.i*. allele (5'-CCAAGAACTTCTGCTAGAC-3' and 5'-TGGCAACTTGTCTAGGGA-3')[7], *Ecad *wt and floxed allele (5'-CTTATACCGCTCGAGAGCCGGA-3') (5'-GTGTCCCTCCAAATCCGATA-3') [11] and p53 floxed allele (5'-CTACCTGAAGACCAAGAAGG-3') (5'-TGGAGGATATGGACCCTATG-3')[14]. The *WAP::Cre *transgene was detected using the following primers (5'-TAGAGCTGTGCCAGCCTCTTC-3') and (5'-CATCACTCGTTGCATCG ACC-3') [12].

### Histology

Whole mount staining of inguinal mammary glands was performed as previously described [15]. Briefly, mammary glands were fixed for 2 to 4 hours in Carnoy's fixative (6 parts 100% ethanol, 3 parts chloroform, 1 part acetic acid). The fixed glands were de-fatted in acetone for 2 hours, stained with carmine solution during the night, and rinsed in tap water. After dehydration, the samples were embedded in paraffin and photographed with a Nikon DXM1200f.

The left thoracic mammary glands were dissected, fixed, dehydrated, embedded in paraffin, and sectioned at 7 μm. Sections were dewaxed, rehydrated, and stained with hematoxylin (#H3136) and eosin (#212954), Masson trichome (#HT15), and Oil Red O [16] (#O0625, all from Sigma Aldrich, München Germany). The stained paraffin slides were photographed with a Zeiss Axiocam.

### Immunohistochemistry, immunofluorescence, and antibodies

Immunohistochemistry (IHC) on paraffin sections of the thoracic mammary gland was performed as described previously [17]. The following antibodies were used. Mouse anti-N-cadherin (#610921), mouse monoclonal anti-E-cadherin (#610182), mouse anti-Stat3 (#610189, all from BD Transduction Laboratories, Heidelberg, Germany). Affinity-purified rabbit anti-gp84 antibody against the extracellular domain of E-cadherin [18], anti-Vimentin monoclonal antibody (#11-254-C100, Exbio/HiSS, Freiburg, Germany,), mouse monoclonal anti-p53 (#PAb421, Enzo, Lörrach, Germany. Rabbit monoclonal anti-Stat5 antibody (,#9358), anti-Erk1/2 antibody (#9102), anti-cleaved Caspase 3 (#9664s), anti-phospho-Erk1/2 antibody (#9101), rabbit monoclonal anti-phospho-Stat3 antibody (#9145, all from Cell Signaling/NEB, Frankfurt, Germany), Anti-CFTR antibody (#SC-10747), anti-WAP antibody (#SC-14832), anti-Snail1 antibody (#SC-10433), anti-MTA1 (#SC-10813, all from Santa Cruz, Heidelberg, Germany). Monoclonal anti N-cadherin (#C3865, Sigma Aldrich, München, Germany), anti-PCNA (#M0879, Dako, Hamburg, Germany), Anti-Sox2 (#245610) and anti-GAPDH antibody (#CB1001, all from Calbiochem/Merck, Darmstadt, Germany).

For mouse and rabbit antibodies, the DAKO Envision+ System HRP was used to amplify the signals (#K4000 and #K4002, DakoCytomation, Hamburg, Germany). For rat and goat antibodies, secondary peroxidase-conjugated anti-rat and anti-goat IgG antibodies (Jackson ImmunoResearch Laboratories, West Grove, USA) were used, respectively. Staining of paraffin sections was visualized with DAB Peroxidase Substrate (#D-4293, Sigma, München, Germany). For immunofluorescence, secondary fluorochrome-conjugated antibodies (Alexa488 and 583) were used (Molecular Probes/Invitrogene, Darmstadt, Germany). The stained paraffin slides were photographed with a Axiocam (Zeiss, Göttingen, Germany) and SP2 UV (Leica, Wetzlar, Germany).

### Western blot analysis

The preparation of protein extracts for Western Blot analysis has been described previously [19]. Briefly, 2 mg of fresh or frozen tissue was homogenized on ice in 2 ml of lysis buffer (#R0891, Fermentas, St. Leon-Rot, Germany) including the phosphatase inhibitor phenylmethyl sulfonyl fluoride (#10837091001, Roche, Mannheim, Germany) and protease inhibitors (Complete, #11836153001, Roche, Mannheim, Germany). The lysates were cleared by centrifugation at 14,000 g for 10 minutes at 4°C, subjected to SDS-PAGE, and transferred to a Polyvinylidenfluorid (PVDF) membrane. Membranes were probed with primary antibodies. Specific binding was detected with horseradish-peroxidase (HRP)-conjugated secondary antibodies (Jackson ImmunoResearch Laboratories, West Grove, USA) and visualized with ECL Plus (#RNP2132, Amersham/GE Healthcare, Freiburg, Germany,).

### *In vitro *organ culture

For application of the Fgf inhibitor SU5402 (#572630, Calbiochem/Merck, Darmstadt, Germany).

mammary glands were isolated and transferred in DMEM medium (#41966, Invitrogen, Darmstadt, Germany) in the absence of serum, either supplemented with 40 μM SU5402 dissolved in dimethyl sulfoxide (DMSO) or the corresponding amount of DMSO as a control. After 24 hours of incubation at 37°C and 10% CO2 the tissue was treated as described above.

## Results

### N-cadk.i. mice lose lactation capacity

As a first approach, the different mutant and control mice were examined for their lactation capacity. For this, pups were weighed each day, starting at the day of birth. Pups from WAP::Cre;Ecad^fl/+ ^(referred to as control) females gained weight progressively until the end of the lactation period at day 18. The end of lactation was followed by the transition to solid food uptake and was accompanied by a transient stagnation of body weight (Additional File 1). In contrast, pups from WAP::Cre;Ecad^Ncad/fl ^(referred to as Ncadk.i.) females died two to three days after birth. We could not detect any signs of milk in the stomach of these pups, indicating that lactation was impaired before the offspring were born (data not shown). WAP::Cre;Ecad^fl/fl ^(referred to as Ecadk.o.) females also showed a defect in lactation capacity. In contrast to Ncadk.i., the mother could feed the offspring for the first ten days and the pups gained weight comparable to control mice. Then, the lactation stopped and the offspring died within two to three days (Additional File 1A). Interestingly, Ncadk.i. females showed a more severe phenotype in comparison to Ecadk.o. animals, as measured by the lactation capacity. This result may indicate that the effects observed in Ncadk.i. mice did not originate from the lack of E-cad. Rather, the presence of N-cad may account for the severe decrease in lactation capacity. For controls, pups were exchanged between Ncadk.i., Ecadk.o., and control animals. Pups of Ncadk.i. and Ecadk.o. mutant females survived and developed normally when fed by control females. Conversely, when pups of control mice were placed with mutant mothers, the pups lost weight progressively and died (data not shown). These results clearly demonstrate that the cause of death is the incapability of Ncadk.i. mutant mice to nurse the offspring.

### The morphology of Ncadk.i. and Ecadk.o. mammary glands is highly disturbed

**Figure 1:**
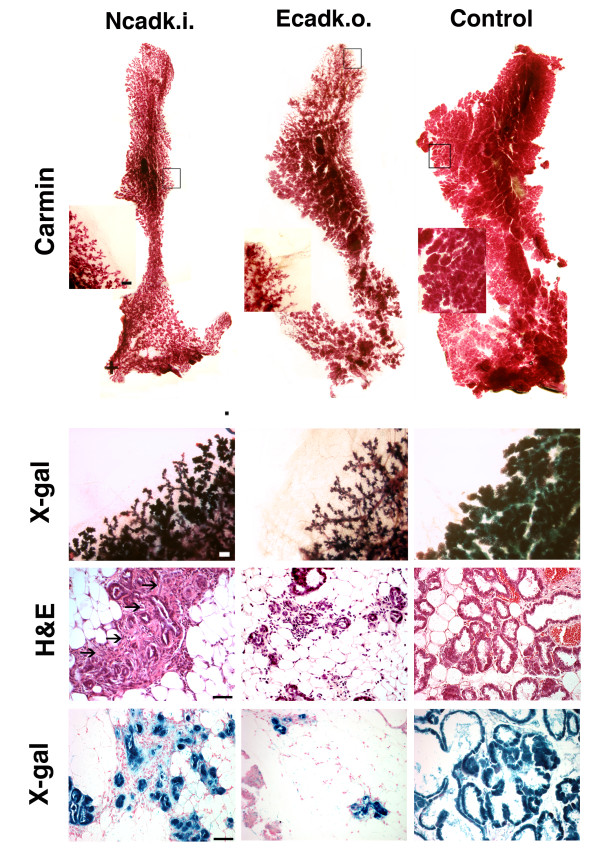
**The morphology of Ncadk.i. and Ecadk.o. mammary glands is profoundly disturbed**. Carmine whole mount staining of Ncadk.i. and Ecadk.o. mammary glands revealed size differences (upper panel) and reduction of lobulo-alveolar structures compared to the controls (see insets in the upper panel). Cre recombination activity was visualized using a ROSA26 reporter and demonstrated the efficiency of the WAP::Cre transgenic line in whole mount X-Gal staining and sections. In H&E-stained sections, the collapsed lobulo-alveolar structures in mutants became apparent. Ncadk.i. mammary glands also showed pronounced accumulation of fibrous tissue around the collapsed alveoli (arrows). Scale bars: Carmine: 1 mm; Carmine insets and X-gal whole mount: 3 mm; sections: 100 μm. Control, WAP::Cre;*Ecad^fl/+ ^*; Ecadk.o., WAP::Cre;*Ecad^fl/fl ^*; H&E: hematoxylin and eosin; Ncadk.i., WAP::Cre;*Ecad^Ncad/fl ^*; ROSA26: reverse orientation splice acceptor 26; WAP::Cre: Whey acidic protein::Cre.

**Figure 2:**
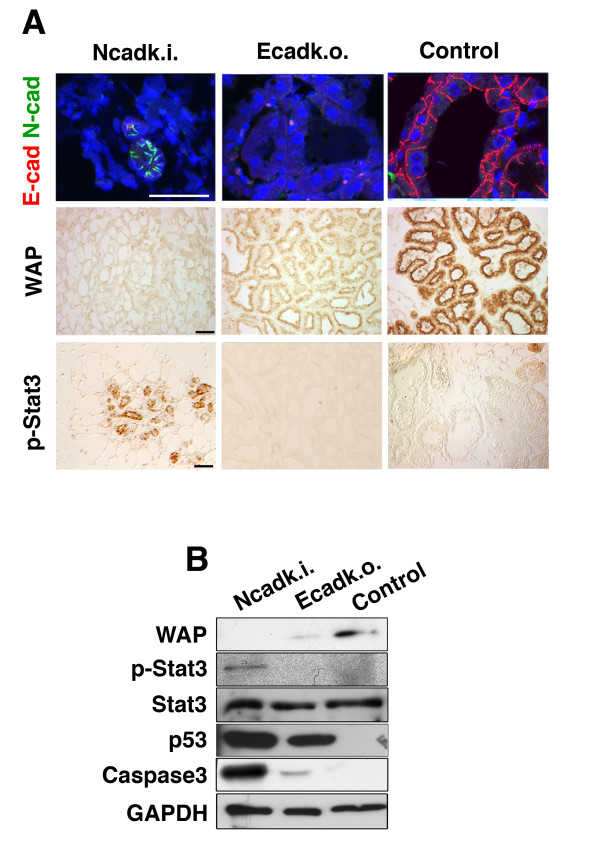
**Ncadk.i. mammary glands exhibit signs of precocious involution**. **(A) **Immunofluorescence of paraffin sections revealed a mislocalization of N-cad in collapsed alveoli of Ncadk.i. mammary glands. No E-cad could be detected in Ecadk.o. alveoli, which looked morphologically normal. In control samples, E-cad but not N-cad, could be found at the basolateral site of alveolar epithelial cells. DAPI was used to visualize nuclei of the cells. The alveoli of Ecadk.o. females show immunoreactivity for WAP while WAP is completely absent in Ncadk.i. alveoli. P-Stat3, a key player in the involution process, is highly upregulated in Ncadk.i., whereas Ecadk.o. and control did not show any signal for p-Stat3. Scale bar: 100 μm. **(B) **Western Blot analysis of mammary glands on the second day of the second lactation cycle. WAP is highly decreased in Ecadk.o. and completely absent in Ncadk.i. mammary glands compared to control. P-Stat3 is induced in Ncadk.i., indicating involution. P53, an inducer of apoptosis, is highly upregulated in Ncadk.i. and Ecadk.o.; however, cleaved caspase3 was predominantly detected in Ncadk.i. mammary glands. GAPDH was used to ensure the loading of equal amounts of proteins. Control, WAP::Cre;*Ecad^fl/+ ^*; DAPI, 4',6-diamidino-2-phenylindole; E-cad, E-cadherin; Ecadk.o., WAP::Cre;*Ecad^fl/fl^*; GAPDH, Glyceraldehyde 3-phosphate dehydrogenase; N-cad, N-cadherin; Ncadk.i., WAP::Cre;*Ecad^Ncad/fl^*;p53, protein 53; P-Stat3, Phospho Signal transducer and activator of transcription 3; WAP, Whey acidic protein.

Since the recombination efficiency of the WAP::Cre line increases substantially in the second lactation cycle compared to the first (data not shown [12]), we performed the analyses of the mammary glands (MG) at the third day of the second lactation cycle. Figure 1 shows MG from Ncadk.i., Ecadk.o., and control animals. In each case, the left anterior thoracic MG was taken for comparison. Whole mount carmine staining revealed that the MG from Ncadk.i. and Ecadk.o. were smaller in size and contained fewer lobulo-alveolar structures as compared to controls (Figure 1, upper panel and Additional File 1B). H&E staining of tissue sections revealed that the morphology of Ncadk.i. and Ecadk.o. MG was highly disturbed (Figure 1). Both Ncadk.i. and Ecadk.o. showed collapsed alveolar structures. The recombination activity of WAP::Cre was monitored by introducing a ROSA26 reporter allele [20]. Whole mount X-Gal staining and sections demonstrated the efficient Cre recombination activity in mutant and control samples resulting in highly decreased E-cad levels in mutants (Figure 1, lower panel, Additional File 2). Immunohistological analysis of Ncadk.i. MG revealed a mislocalization of N-cad at the luminal site of epithelial cells in collapsed alveoli (Figure 2A). In comparison, intact alveoli of control MG showed E-cad at the basolateral site of alveolar epithelial cells (Figure 2A). Interestingly, the remaining intact alveoli in Ecadk.o. MG were negative for E-cad (Figure 2A). This observation suggests that the lack of E-cad did not immediately lead to the collapse of all alveolar structures. In contrast, the complete alveolar architecture was affected in Ncadk.i. MG. The comparative immunohistological analyses are in agreement with the different lactation capacities of Ncadk.i. and Ecadk.o. reported above. Whey acidic protein (WAP), a component of the milk and a marker for milk productivity, was completely absent in Ncadk.i. and decreased in Ecadk.o. compared to control (Figure 2A, B). Surprisingly, Phospho-Stat3 (p-Stat3), a key player in the involution process [21], is strongly activated in Ncadk.i. alveolar epithelial cells. In contrast, Ecadk.o. and the control did not show the activation of this involution marker (Figure 2A, B). In accordance with the activation of p-Stat3, its downstream components p53 and cleaved caspase3 (which initiate the involution process) are highly activated in Ncadk.i. MG (Figure 2B). Remarkably, p53 is also activated in Ecadk.o MG, although p-Stat3 is not detectable. These data may indicate that p53 can be activated by different pathways.

### Deletion of one p53 allele rescues the lactation capacity of Ncadk.i. but not of Ecadk.o. females

**Figure 3:**
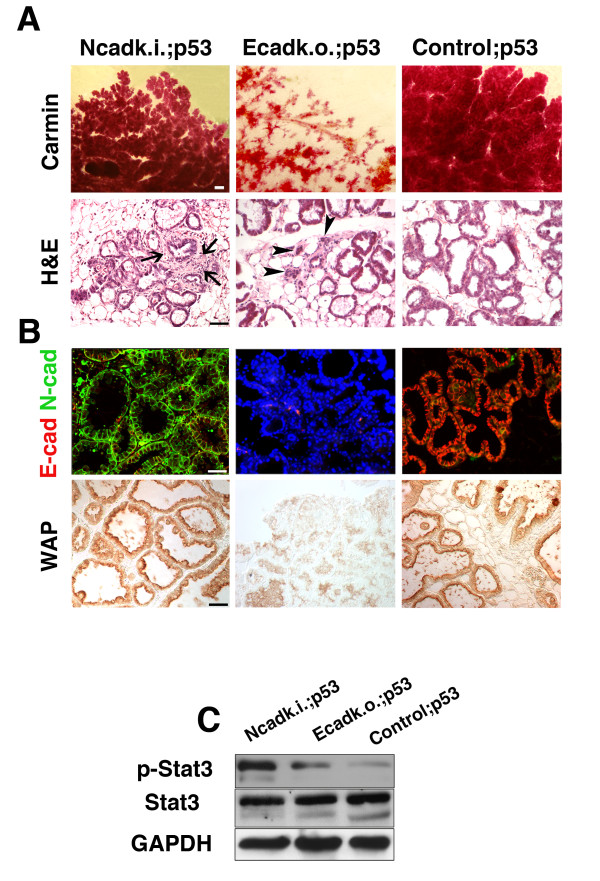
**Morphology and function of Ncadk.i.;p53 mammary glands are comparable to control samples**. **(A) **Whole mount carmine staining of Ncadk.i.;p53 mammary glands showed comparable lobulo-alveolar structures to control;p53, while Ecadk.o.;p53 mammary glands possess only poorly developed structures. This observation was confirmed by H&E-stained paraffin sections showing intact alveoli in Ncadk.i.;p53 and control;p53 mammary glands and collapsed lobulo-alveolar structures in Ecadk.o.;p53 mammary glands (arrowheads). The accumulation of fibrous tissue surrounding the alveoli in Ncadk.i.;p53 was still present (arrows). Scale bar: Carmin: 3 mm; H&E: 100 μm **(B) **Immunofluorescence on paraffin sections revealed basolateral localization of N-cad in the alveoli of Ncadk.i.p53 mammary glands, resembling E-cad staining in control;p53 samples. DAPI was used to visualize the nuclei in Ecadk.o.;p53 sections, which were negative for E-cad. The detection of WAP was used to verify the function of the alveoli. Ncadk.i.p53 and control;p53 showed similar WAP levels, while Ecadk.o.p53 exhibited highly decreased levels. Scale bar: 100 μm **(C) **Western Blot analysis of Ncadk.i.p53, Ecadk.o.;p53, and control;p53 mammary gland lysates. Notably, Ncadk.i.p53 shows a constitutive activation of p-Stat3. control;p53, WAP::Cre; *Ecad^fl/+^*; DAPI, 4',6-diamidino-2-phenylindole; E-cad, E-cadherin; Ecadk.o.;p53, WAP::Cre;*Ecad^fl/fl^*;*p53^fl/+^*; H&E, Hematoxylin and Eosin; N-cad, N-cadherin; Ncadk.i.;p53, WAP::Cre;*Ecad^Ncad/fl^*;*p53^fl/+^*; *p53^fl/+^*; WAP, Whey acidic protein.

Downstream events of Stat3-mediated involution include p53-dependent apoptosis of epithelial cells in the MG. Conditional heterozygous deletion of p53 was performed by introducing p53 floxed alleles in Ncadk.i., Ecadk.o, and control females to eventually block the precocious involution observed in Ncadk.i. MG (Additional File 3). Strikingly, Ncadk.i. mice carrying only one intact allele of p53 (WAP::Cre;Ecad^Ncad/fl^;p53^fl/+^, referred to as Ncadk.i.;p53) exhibited a lactation capacity comparable to control mice (WAP::Cre;Ecad^fl/+^;p53^fl/+^, referred to as control;p53) (Additional File 4A). In contrast, the lactation capacity of Ecadk.o.;p53 females (WAP::Cre;Ecad^fl/fl^;p53^fl/+^) was not rescued by the deletion of one p53 allele (Additional File 4A). The morphologies of MG from Ncadk.i.;p53 and control;p53 animals on the third day of the second lactation cycle visualized by carmine staining were remarkably similar (Figure 3A). Both showed well-developed lobulo-alveolar structures. In contrast, Ecadk.o.;p53 exhibited few lobulo-alveolar structures (Figure 3A and Additional File 4B). H&E staining of sections from Ncadk.i.;p53 MG showed normal tissue integrity, similar to the control. In contrast, in Ecadk.o.;p53, the lobulo-alveolar structure appeared collapsed, leaving clusters of epithelial cords with small lumina (Figure 3A, arrowheads).

From these results, we concluded that removal of one p53 allele is beneficial for the function and morphology of Ncadk.i. MG. Immunostaining revealed that N-cad was properly localized at the basolateral site of the MG epithelium, comparable to E-cad in control;p53 (Figure 3B). Milk production, visualized by WAP staining, was fully functional in Ncadk.i.;p53 and control;p53, whereas Ecadk.o.;p53 MG did not exhibit the presence of WAP (Figure 3B).

Intriguingly, we detected high levels of p-Stat3 in Ncadk.i.;p53 MG. P-Stat3 is known to initiate the involution process by activating p53-dependent apoptosis in wild type MG (Figure 3C). The absence of apoptosis in Ncadk.i.;p53 MG indicates that p53 acts downstream of p-Stat3 signaling and is central in controlling the apoptotic process in involution. Moreover, the action of p53 in involution seems to be highly dosage-dependent because apoptosis was avoided by the deletion of only one p53 allele in Ncadk.i.;p53 MG. In contrast, the heterozygous deletion of p53 in Ecadk.o. mice could not rescue the Ecadk.o. phenotype.

### Ncadk.i. and Ncadk.i.;p53 females develop fibrosis and cysts in the MG with increasing lactation cycles

**Figure 4:**
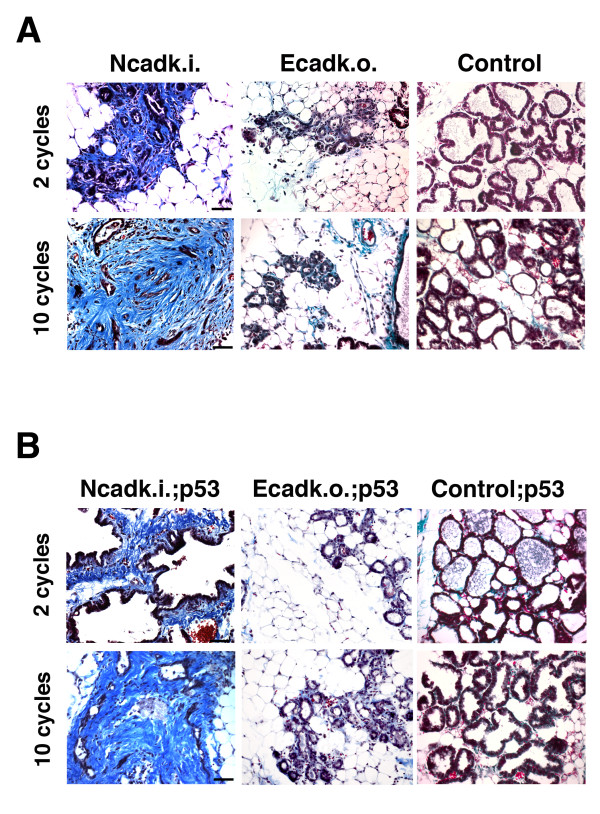
**Accumulation of fibrous tissue in the mammary gland of Ncadk**.i. and Ncadk.i.;p53 mice. Trichrome staining of paraffin sections was used to visualize the accumulation of fibrous tissue in **(A) **Ncadk.i. and **(B) **Ncadk.i.;p53 mammary glands in the second and tenth lactation cycles. The pronounced deposit of collagen fibers in the second lactation cycle became markedly increased in females after ten lactation cycles. Mammary glands from Ecadk.o. and Ecadk.o.;p53 females showed only minor signs of fibrosis in comparison to the controls. Scale bar: 100 μm. Ecadk.o.;p53, WAP::Cre;*Ecad^fl/fl^*;*p53^fl/+^*; Ecadk.o., WAP::Cre;*Ecad^fl/fl^*; Ncadk.i., WAP::Cre;*Ecad^Ncad/fl^*; Ncadk.i.;p53, WAP::Cre;*Ecad^Ncad/fl^*;*p53^fl/+^*.

**Figure 5:**
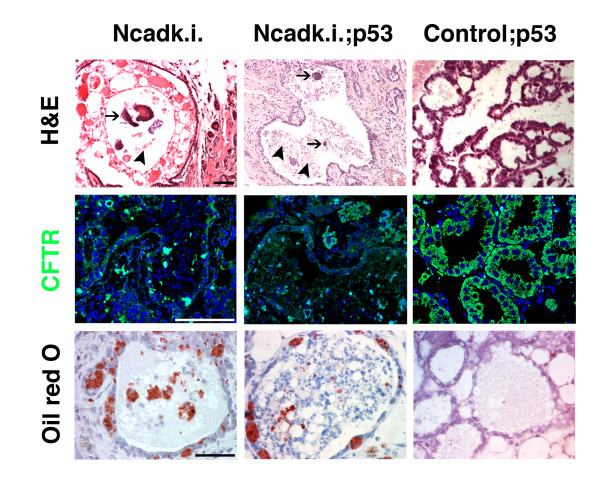
**Cyst formation in Ncadk.i. and Ncadk.i.;p53 mammary glands**. With increasing lactation cycles, the accumulation of fibrous tissue was accompanied by the formation of cysts. After ten lactation cycles, H&E-stained paraffin sections showed cyst formation in both Ncadk.i. and Ncadk.i.;p53 mammary glands, while no cysts were observed in controls. Premalignant marks, such as calcification (arrows) and accumulation of necrotic material (arrowheads), were detected in both lines. Cyst formation was accompanied by a downregulation of CFTR. Oil Red staining showed the accumulation of lipids in alveolar epithelial cells in Ncadki. and Ncadk.i;p53 mammary glands. Scale bar: 100 μm. CFTR, Cystic Fibrosis Transmembrane Conductance Regulator; H&E, Hematoxylin and Eosin; Ncadk.i., WAP::Cre;*Ecad^Ncad/fl^*; Ncadk.i.;p53, WAP::Cre;*Ecad^Ncad/fl^*;*p53^fl/+^*.

Hematoxylin and eosin staining of Ncadk.i. (Figure 1, arrows) and Ncadk.i.;p53 (Figure 3A, arrows) already suggested increased fibrotic accumulations in the MG. To further investigate this phenomenon, we performed Trichrome staining. Indeed, in the second lactation cycle, Ncadk.i. and Ncadk.i.;p53 MG showed an accumulation of blue-stained collagenic fibers around collapsed and intact alveoli (Figure 4A and 4B). With increasing lactation cycles, the accumulation of fibrotic tissue became increasingly apparent and predominant. After 10 lactation cycles (Figure 4A and 4B), fibrotic tissue appeared to displace the alveolar morphology in Ncadk.i.;p53 MG, which ultimately led to impaired lactation. No increased fibrosis was observed in Ecadk.o. and Ecadk.o.;p53 mice, nor in controls (Figure 4A and 4B). Fibrosis is often accompanied by the formation of cyst-like structures, which is only observed in Ncadk.i. and Ncadk.i.;p53 animals (Figure 5). Cysts became apparent in Ncadk.i.;p53 several lactation cycles earlier as compared to Ncadk.i. animals. These observations indicate that N-cad may have an inductive role in cyst formation that is enhanced by the lack of p53. Cystic fibrosis transmembrane conductance regulator (CFTR) is an ABC transporter-class ion channel that transports chloride and thiocyanate ions across epithelial cell membranes. Mutations of the CFTR gene in humans affect functioning of the chloride ion channels in cell membranes, leading to cystic fibrosis [22,23]. In addition, CFTR is required in intestinal Caco cells for proper lipid transport through the cell membranes [24,25]. In control;p53 MG, CFTR is expressed throughout the alveolar epithelial cells (Figure 5). However, in Ncadk.i. and Ncadk.i.;p53, CFTR expression is greatly reduced, which may perturb lipid secretion as visualized by Oil Red staining (Figure 5). Lipid droplets were found accumulated in alveolar epithelial cells of Ncadk.i. and Ncadk.i;p53 MG, but not in control;p53 (Figure 5) or in Ecadk.o. and Ecadk.o.;p53 MG (data not shown). From these results, we concluded that cyst formation is accompanied by reduced CFTR levels in the MG of Ncadk.i. and Ncadk.i.;p53 as observed in human cystic fibrosis. However, whether the lack of CFTR contributes directly to the formation of cysts needs to be elucidated.

### Alveolar epithelial cells of the MG in Ncadk.i. mice upregulate markers for epithelial-mesenchymal transition (EMT) and gain migratory properties

**Figure 6:**
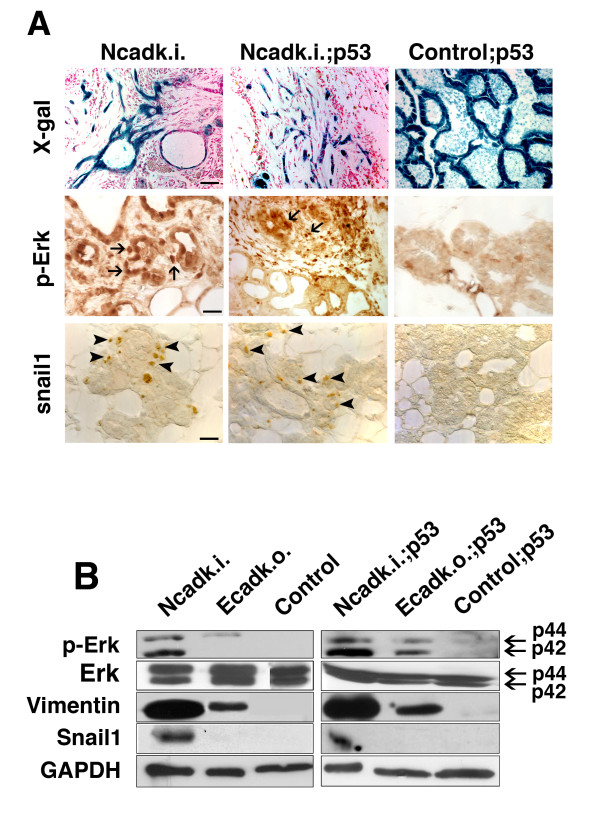
**Ncadk.i. alveolar epithelial cells exhibit signs of epithelial-mesenchymal transition (EMT)**. **(A) **The ROSA26 reporter allele was used to trace recombined alveolar epithelial cells. LacZ expression, visualized by X-gal staining, indicates Cre activity which normally is restricted to alveolar epithelial cells as shown in the control;p53 sample. Many LacZ-positive epithelial cells in Ncadk.i. and Ncadk.i.;p53 mammary glands changed their cell shape, migrated outside the alveoli, and were found in the stromal compartment. This process was accompanied by the activation of p-Erk and the upregulation of Snail1. Scale bar: 100 μm. **(B) **Western Blot analysis revealed the upregulation of the mesenchymal marker Vimentin in Ncadk.i. and Ncadk.i.;p53 mammary glands. Notably, Vimentin, but not Snail, is also upregulated in Ecadk.o. and Ecadk.o.;p53. GAPDH was used for the loading control. control;p53, WAP::Cre; *Ecad^fl/+^*; Ncadk.i., WAP::Cre;*Ecad^Ncad/fl^*; GAPDH, Glyceraldehyde 3-phosphate dehydrogenase; Ncadk.i.;p53, WAP::Cre;*Ecad^Ncad/fl^*;*p53^fl/+^*; p-Erk, phospho-extracellular signal-regulated kinase; ROSA26, reverse orientation splice acceptor 26; X-gal, 5-bromo-4-chloro-indolyl-galactopyranoside.

By tracing recombined cells with the ROSA26 reporter allele, we frequently observed alveolar epithelial cells leaving the epithelial cell layer and accumulating in the stroma in Ncadk.i. and Ncadk.i.;p53 females with advanced lactation cycles (Figure 6A). These data suggested that alveolar epithelial cells in Ncadk.i. and Ncadk.i.;p53 MG undergo epithelial-mesenchymal transition (EMT) to gain migratory properties. This idea was supported by the fact that the mesenchymal marker Vimentin was highly upregulated in the MG of these mice (Figure 6B). Transcription factors of the Snail family have been shown to be essential for the initiation of EMT. We found Snail1 induced in epithelial cells of Ncadk.i. and Ncadk.i.;p53 MG (arrowheads in Figure 6A), whereas no Snail1 was detected in control samples (Figure 6A and 6B). Since Snail1 is a direct target of Fgf signaling [26,27], we examined the phosphorylation status of Fgf receptor 1 (Fgfr1) as well as the downstream effector of Fgf signaling, Erk. Active Fgf signaling marked by phosphorylated Fgfr1 (p-Fgfr1) was found in Ncadk.i. and Ncadk.i;p53 MG (Additional File 5). Accordingly, phosphorylated Erk (p-Erk) was also detected in these tissues (Figure 6A arrows, Figure 6B). Whereas control samples showed a complete lack of active Fgf signaling, Ecadk.o. and Ecadk.o.;p53 MG exhibit moderate p-Erk levels in the absence of p-Fgfr (Figure 6B and Additional File 5). These data suggest that N-cad plays an inductive role in turning on the Fgf signaling pathway in Ncadk.i. and Ncadk.i.;p53 MG. To obtain further evidence for these results we performed *in vitro *organ culture. Freshly isolated Ncadk.i.;p53 MG were treated with a Fgf signaling inhibitor, SU5402 which inhibits specifically the tyrosin kinase activity of Fgfr1 [28]. A corresponding amount of DMSO was used as a control. As expected, the protein levels of p-Stat3, p-Erk and Snail1 were reduced upon SU5402 treatment compared with the control confirming our *in vivo *observation (Additional File 6). Interestingly, the detection of EMT markers and the observation of increased motility of alveolar epithelial cells happened in a temporally distinct manner. Snail and p-Erk were found even in early lactation cycles, but migratory epithelial cells were only visible from the eighth lactation cycle onwards (not shown). These data may indicate that alveolar epithelial cells require additional stimuli to achieve migratory properties. Ecadk.o. and Ecadk.o.;p53 MG do not show any migrating alveolar cells (Additional File 7). This suggests that the lack of E-cad in alveolar epithelial cells is not sufficient to induce EMT and gain migratory properties.

### Transition from benign to malignant structures and tumor formation

**Figure 7:**
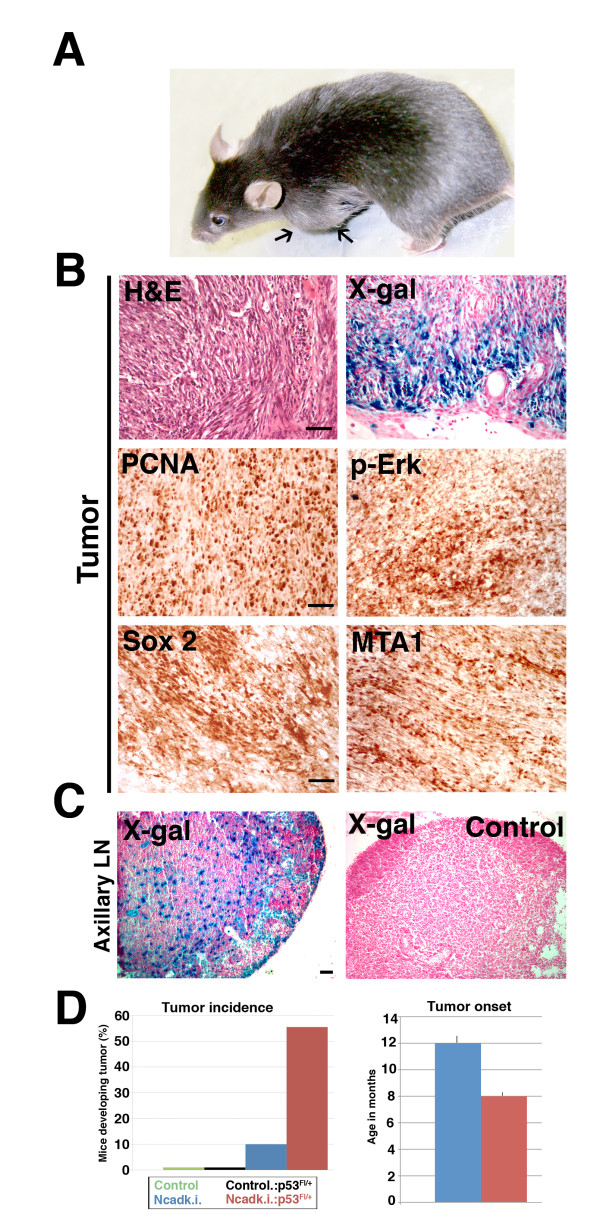
**Ncadk.i. and Ncadk.i.;p53 females develop malignant and highly invasive tumors**. **(A) **Ncadk.i. and Ncadk.i.;p53 females developed large tumors (arrows) after numerous lactation cycles that were always found in the left thoracic mammary gland. **(B) **H&E staining of tumor sections revealed poorly differentiated cells. X-gal staining identified many transformed alveolar epithelial cells, which contribute to the tumor structure. Immunohistochemistry on paraffin sections revealed that tumor cells are highly proliferative, indicated by PCNA staining, and express high levels of p-Erk, Sox2, and the metastatic marker MTA1. Scale bar: 100 μm **(C) **X-gal staining traced alveolar epithelial cells derived from the tumor in the axillary lymph node, demonstrating the metastatic capacity of the tumor cells. No LacZ-positive cells were found in control lymph nodes. Scale bar: 100 μm. **(D) **Heterozygous deletion of p53 enhances the tumorigenic potential of N-cad. At least nine animals were analyzed for tumor incidence and tumor onset. Ten percent of Ncadk.i. mice develop mammary tumors. This number increases to more than 50% upon additional heterozygous deletion of p53 (left panel). The average age of tumor onset is decreased in Ncadk.i.;p53 compared to Ncadk.i. mice (right panel). H&E, Hematoxylin and Eosin; MTA1, Metastatic tumor antigen1; Ncadk.i., WAP::Cre;*Ecad^Ncad/fl^*; Ncadk.i.;p53, WAP::Cre; *Ecad^Ncad/fl^*;*p53^fl/+^*; p53, protein 53; p-Erk, phospho-extracellular signal-regulated kinase; Sox2, SRY (sex determining region Y)-box 2; X-gal, 5-bromo-4-chloro-indolyl-galactopyranoside.

In aged Ncadk.i and Ncadk.i.;p53 mice with numerous lactation cycles, we observed a histological transition from benign to malignant structures. These structures included calcification (Figure 5, arrows), accumulation of necrotic material inside the cysts (Figure 5, arrowheads), ductal ectasia, lymphocytic ductitis, and ductal hyperplasia (data not shown). Large tumors were observed in the left thoracic mammary gland of Ncadk.i. and Ncadk.i.;p53 females (Figure 7A, arrows). In accordance with the well-known role of p53 in tumor suppression, increased tumor incidence was found in Ncadk.i;p53 (five out of nine) as compared to Ncadk.i. (one out of ten) animals (Figure 7D). The reduction of p53 also decreases the time of tumor onset in Ncadk.i. mice (Figure 7D). Strikingly, Ecadk.o., Ecadk.o.;p53, and WAP::Cre;p53 females did not show tumor formation even in aged mice (data not shown). Histological analysis of tumors from Ncadk.i.;p53 animals showed poorly differentiated cells and a sarcoma-like morphology (Figure 7B, upper left panel). Many cells in the tumor were positive for LacZ, indicating that these cells are transformed alveolar epithelial cells that contribute to the tumor. Moreover, Proliferating cell nuclear antigene (PCNA) staining revealed that nearly all of the cells were in a proliferative state (Figure 7B). As shown above, we supposed that p-Erk signaling initiated EMT in alveolar epithelial cells. We found many cells inside the tumor that still maintained p-Erk signaling, suggesting that this pathway supports tumor growth (Figure 7B). Sox2 has been shown to be involved in breast tumorigenesis [29]. We detected abundant Sox2 expression in the tumor (Figure 7B). In addition, we found extensive staining for metastasis-associated protein 1 **(**MTA1). Expression of MTA1 has been correlated with metastatic potential in two types of carcinomas [30,31] (Figure 7B). In line with the high expression of MTA1, we found LacZ-positive cells in the left axillary lymph node of tumor-afflicted mice (Figure 7C). These data clearly demonstrate that epithelial cells of the mammary gland are transformed and highly invasive in Ncadk.i.;p53 mice. Interestingly, no tumors were observed in Ecadk.o.;p53 or WAP::Cre p53, even in females with numerous lactation cycles (data not shown). Although the pivotal role of p53 in involution and tumor formation has been reported previously [32,33], these results demonstrate the synergistic potential of N-cad together with p53 in promoting tumor formation in the mammary gland.

## Discussion

The morphology and function of lobulo-alveolar structures in the mammary gland are highly dependent on the expression of E-cad [34]. Female mammary glands deficient for E-cad lose milk productivity and exhibit collapsed lobulo-alveolar structures with signs of premature involution and apoptosis [11]. According to these results, E-cad has been proposed to be a survival factor for the epithelial cells of the mammary gland [11]. To explore the specific functions of E-cad in comparison to N-cad *in vivo*, we made use of a knock-in mouse line in which N-cad is expressed under the regulatory control of the E-cad locus [7]. In our approach, we combined the Ncadk.i. line with the conditional ablation of the remaining E-cad allele. We directed the conditional ablation to the alveoli of the mammary gland using WAP::Cre, which efficiently recombined the remaining E-cad floxed allele starting at the late pregnancy stage.

In the first part of our study, we analyzed the lactation capacity of Ncadk.i., Ecadk.o., and control animals and found some unexpected results. From our previous work [11], we had anticipated that the Ecadk.o. mice would exhibit a more drastic phenotype, and we had hoped that the presence of the Ncadk.i. allele would be beneficial for the structure and function of the mammary gland. But the opposite is the case, in that complete feeding impairment was observed only in Ncadk.i. mice. The fact that Ecadk.o. females can nurse their offspring for about 10 days may be explained by the relative late induction of Cre expression by WAP::Cre, compared to the early Cre expression in the MMTV::Cre transgenic line, which was used previously [11]. In addition, the known stability of the E-cad protein may preserve some alveolar structures even when the E-cad floxed allele is already recombined. In this respect we analyzed tight junctional proteins as Occludin and ZO1 which may account for the stabilization of the epithelial cell layer in the absence of E-cad. However, both factors are found to be more weakly expressed and mislocalized (data not shown). This is in line with previous reports which found that E-cad regulates tight junctional complexes in epithelial cells [35]. Histological analysis of Ncadk.i. mammary glands revealed collapsed lobular-alveolar structures, a feature which resembles the involution process. Indeed, we found that p-Stat3, an involution marker, was induced in Ncadk.i. MG. In contrast, p-Stat3 was not upregulated in Ecadk.o. alveoli, indicating that the involution program was not immediately triggered despite elevated levels of p53. Rather, the sole expression of N-cad induced precocious involution. Our results differ from those of a previous report where a transgenic mouse was generated to overexpress N-cad in the mammary gland epithelium in the presence of E-cad [36]. In these mice the mammary glands appeared normal and no tumors arose spontaneously. Accordingly, Ncadk.i./+ mice do not show any abnormality and develop normally [7]. In comparison to our results this clearly underlines the important role of E-cad in maintaining tissue integrity and its tumor suppressor function, even in the presence of N-cad. In addition this shows that E-cad can neutralize the action of N-cad in MG epithelial cells *in vivo*, an observation which was made before in cancer cell lines [37].

Stat3 is the key player in the involution process [21]. Its activation depends mainly on receptor tyrosine kinase (RTK) signaling, in particular, on the Fgf signaling pathway [26,38]. Fgf signaling has previously been shown to be able to be activated by the physical interaction of N-cad and Fgf receptor *in vitro *[39]. Our *in vivo *results are in agreement with these observations, as Ncadk.i MG showed high levels of p-Fgfr, p-Erk, and the upregulation of Fgf target genes like Stat3 and Snail1. At the onset of involution, p-Stat3 is known to induce the upregulation of pro-apoptotic factors, such as p53, in mammary glands [40]. Thus, we performed heterozygous deletion of p53 to eventually interfere with the precocious involution and apoptosis. Surprisingly, the deletion of one p53 allele completely rescued the lobulo-alveolar structure and the lactation capacity of Ncadk.i. mice, but not of Ecadk.o. mice. This result is remarkable considering the unique role of E-cad and the interaction with the epidermal growth factor receptor, which has been proposed to be essential for mammary gland function [41,42]. Here we show that N-cad can replace E-cad as an adhesion molecule for the integrity of the mammary epithelial cell layer for at least 5 lactation cycles. However, the adhesion function of N-cad becomes apparent only if the p53 level is reduced. These data suggest that the functions of cadherins and p53 are closely linked. Furthermore, the data show the pivotal role of p53 in regulating the balance between apoptosis and proliferation in a highly dosage-dependent manner. A 50% reduction of p53 was sufficient to rescue the cells from apoptosis. The expression of N-cad shifted this balance towards apoptosis, most likely due to the activation of the Fgf pathway. The constitutive activation of Fgf signaling may also be the cause of the increased fibrosis and cyst formation that is observed in Ncadk.i. mice after several lactation cycles. The formation of cysts is closely associated with fibrotic processes [43]. We suppose that increased fibrosis in the mammary gland imposes mechanical pressure on ducts and alveoli which then results in cyst formation. The pathological phenotype of our mice closely resembles fibrocystic mastopathy (FM), which is a common human disorder affecting 30% to 60% of the world's female population [44]. FM is characterized by a massive accumulation of fibrous tissue at the interstitial spaces and the formation of cysts [45]. Mutations in the CFTR gene have been shown to promote FM [23]. Interestingly, previous results have suggested that p-Erk mediates the downregulation of CFTR expression [46]. The activation of p-Erk signaling, accompanied by CFTR downregulation, is observed in the mouse model shown here, suggesting an important role for Fgf signaling in FM. CFTR has profound effects on lipid metabolism [47], and its downregulation is implicated in increased lipid synthesis and accumulation in intestinal Caco cells [24]. Similarly, the loss of CFTR in our mice resulted in lipid accumulation in mammary epithelial cells.

In addition, our mouse model showed premalignant features as reported for FM, including calcifications inside the alveoli [48], collagen fiber deposition [49], lymphocytic infiltrations, and ductal hyperplasia [50]. The transition from benign to malignant structures in our mouse model is potentiated by the heterozygous p53 deletion, which is in accordance with the well-established role of p53 as a tumor suppressor [51]. The fact that Ecadk.o., Ecadk.o.;p53, and WAP::Cre;p53 females did not show any tumor formation may be explained by the genetic background of the mice used in this study which is predominantly C57Bl6. This strain has been shown to have a significantly lower susceptibility to mammary tumorigenesis compared to other inbred strains in particular Balb/c [52,53]. Furthermore, it becomes evident that temporal and spatial differences of conditional approaches play a pivotal role. For example, the conditional deletion of p53 and E-cad in alveolar progenitor cells leads to invasive lobular carcinoma [13], whereas mature alveolar cells do not show any oncogenic transformation lacking p53 and E-cad as seen in our approach. A prerequisite for the development of invasive tumors is that epithelial cells undergo EMT, which is a process in which epithelial cells lose cell adhesion contacts, upregulate mesenchymal genes, and gain cell motility [54]. EMT plays fundamental roles in the development and metastasis of epithelial tumors [55]. Using ROSA26 reporter mice, we were able to trace alveolar epithelial cells. We clearly show that many cells in Ncadk.i.;p53 mammary glands leave the epithelial cell cluster and migrate through the stroma. On the molecular level, we have detected the upregulation of several genes that are implicated in EMT, including Vimentin, Snail1 and p-Erk [56]. Snail1 binds to E-Boxes of the E-cad promoter and induces the downregulation of E-cad [57]. N-cad expression from the Ncadk.i. allele should also be affected since it is driven by the endogenous E-cad promoter. So far, we could not determine the upregulation of endogenous N-cad which is another important event in EMT in the transformed cells because the discrimination between endogenous and knocked-in N-cad mRNA is technically challenging. EMT is most likely initiated in alveolar epithelial cells of the Ncadk.i. mice by constitutive Fgf signaling, which ultimately leads to the appearance of malignant tumors. Molecular analysis of the tumors revealed an enhanced expression of Sox2 and Stat3, which depend on Fgf signaling [30,39], and of the metastasis associated protein (MTA1), which is regulated by Stat3 [31]. Altogether, these results provide compelling evidence that the tumor initiating cells are transformed alveolar epithelial cells that adopt a mesenchymal fate and form a sarcoma-like tumor.

## Conclusions

Our gene replacement approach demonstrates that N-cad in the absence of E-cad induces constitutively active Fgf signaling and premature involution, which results in massive apoptosis of alveolar cells. Additional deletion of one p53 allele inhibits the pro-apoptotic effect of N-cad and leads to a temporal rescue of morphology and function of alveoli. The deletion of E-cad also leads to cell death of alveolar cells, however, this cannot be rescued by p53 deletion. This indicates that N-cad provides the essential features to maintain tissue architecture and function which implies fully overlapping function of E- and N-cadherin in the alveoli. With increasing age and lactation cycles, fibrosis and cysts are observed in N-cadk.i. mammary glands resulting in the blockage of lactation and finally the appearance of malignant tumors. These effects are potentiated by the heterozygous deletion of p53. The fact that no tumors are observed in p53+/- nor in Ecad,p53 MG suggests a pivotal role of N-cad in the development of breast tumors. Thereby we show that the formation of fibrosis and cysts always precede the development of malignant tumors. Thus, the stromal (fibrotic) compartment in the MG may provide the essential stimuli for the transition of N-cad from its adhesive to an oncogenic function. All these features of our mouse model closely resemble FM in humans and makes this model a valuable tool to investigate the underlying molecular mechanism of fibrosis and cysts and the transition to malignant tumors in the mammary gland.

## Abbreviations

CF: cystic fibrosis; CFTR: cystic fibrosis transmembrane conductance regulator; Control: WAP::Cre;*Ecad^fl/+^*; Control:p53: WAP::Cre;*Ecad^fl/+^*;*p53^fl/+^*; DMSO: dimethylsulfoxid; Ecad k.o.:p53: WAP::Cre;*Ecad^fl/fl^*;*p53^fl/+^*; Ecadk.o: WAP::Cre;*Ecad^fl/fl^*; E-cad: E-cadherin, epithelial cadherin; EMT: epithelial-mesenchymal transition; Erk: extracellular signal-regulated kinase; Fgf: fibroblast growth factor; Fgfr: fibroblast growth factor receptor; FM: fibrocystic mastopathy; GAPDH: glyceraldehyde 3-phosphate dehydrogenase; H&E: hematoxylin and eosin; MMTV: mouse mammary tumor virus; MTA1: metastatic tumor antigen; Ncadk.i.:p53: WAP::Cre;*Ecad^Ncad/fl^*;*p53^fl/+^*; Ncadk.i: WAP::Cre;*Ecad^Ncad/fl^*; N-cad: N-cadherin, neural cadherin; p53: protein 53 or tumor protein p53; PCNA: proliferating cell nuclear antigen; p-Erk: p-Erk phosphorylated active form of Erk; p-Stat3: phosphorylated active form of Stat3; RTK: receptor tyrosine kinase; Sox2: SRY (sex determining region Y)-box 2; Stat3: signal transducer and activator of transcription 3; WAP: whey acidic protein; X-gal: 5-bromo-4-chloro-indolylgalactopyranoside.

## Competing interests

The authors declare that they have no competing interests.

## Authors' contributions

AMK contributed to experimental design, performed experiments, and contributed to data analysis. AH contributed to experimental design, performed experiments, contributed to data analysis and wrote the manuscript. RK contributed to experimental design, contributed to data analysis and wrote the manuscript. All authors read and approved the final manuscript for publication.

## Supplementary Material

Additional file 1 **Ncadk.i. and Ecadk.o. females show impaired lactation capacity and reduced number of intact alveoli**. **(A) **To evaluate the function of the mammary gland, the weight of the pups of the corresponding females were monitored during the normal lactation period at the second lactation cycle. Control pups (green graph) gained weight progressively until reaching the time of weaning (approximately 20 days). Pups from Ecadk.o. females survived only 12 to 13 days (red graph) whereas pups from Ncadk.i. females died only two to three days after birth (blue graph). **(B) **The number of collapsed and intact alveoli was quantified in six individual H&E stained paraffin MG sections of Ncadk.i., Ecadk.o. and control mice from the third day of the second lactation cycle. The percentage of collapsed (red) or intact (blue) alveoli is depicted in relation to the entire amount of alveoli counted on the section (= 100%). Ncadk.i., WAP::Cre;*Ecad^Ncad/fl ^*; Ecadk.o., WAP::Cre;*Ecad^fl/fl ^*; Control, WAP::Cre;*Ecad^fl/+^* Click here for file

Additional file 2 **Protein expression levels of Ecad and Ncad in Ncad.k.i. and Ncad.k.i.:p53 mammary glands**. Western blot analysis of mammary gland protein lysates shows highly reduced protein levels of E-cad in both Ncad.k.i. and Ncad.k.i.:p53 animals as a result of efficient Cre mediated recombination. Ncad is expressed comparable to wildtype Ecad in the control sample. GAPDH was used as a loading control. E-cad, E-cadherin; Ncadk.i., WAP::Cre;*Ecad^Ncad/fl^*; Ncadk.i.;p53, WAP::Cre;*Ecad^Ncad/fl^*;*p53^fl/+^*; N-cad, N-cadherin; GAPDH, Glyceraldehyde 3-phosphate dehydrogenase. Click here for file

Additional file 3 **Heterozygous p53 deletion leads to a marked decrease of active p53**. Western blot analysis of mammary gland protein lysate reveals a approximate 50% decrease of p53 protein level in Ncad.k.i.:p53 compared to Ncad.k.i. GAPDH was used as a loading control. Ncadk.i., WAP::Cre;*Ecad^Ncad/fl^*; Ncadk.i.;p53, WAP::Cre;*Ecad^Ncad/fl^*;*p53^fl/+^*; p53, protein 53; GAPDH, Glyceraldehyde 3-phosphate dehydrogenase. Click here for file

Additional file 4 **The heterozygous deletion of p53 rescues the lactation capacity and the number of intact alveoli of Ncadk.i. but not of Ecadk.o. females**. **(A) **The weights of pups from Ncadk.i.;p53 females (blue graph) monitored during the first four weeks after birth were comparable to the control (green), while the deletion of p53 in Ecadk.o. females had no effect on the survival rate of the offspring (red). **(B) **The number of collapsed and intact alveoli was quantified in six individual H&E stained paraffin MG sections of Ncadk.i., Ncadk.i.;p53 and control;p53 mice from the third day of the second lactation cycle. The percentage of collapsed (red) or intact (blue) alveoli is depicted in relation to the entire amount of alveoli counted on the section (= 100%). Ncadk.i.;p53, WAP::Cre;*Ecad^Ncad/fl^*;*p53^fl/+^*; control;p53, WAP::Cre; *Ecad^fl/+^*; *p53^fl/+^*; Ecadk.o.;p53, WAP::Cre;*Ecad^fl/fl^*;*p53^fl/+^*; Ncadk.i., WAP::Cre;*Ecad^Ncad/fl^*. Click here for file

Additional file 5 **Fgf receptor activation is detected in Ncad.k.i. and Ncad.k.i.:p53 but not in Ecad.k.o. nor Ecad.k.o.:p53 mammary glands**. Western blot analysis of mammary gland protein lysates reveals the presence of p-Fgfr in both Ncad.k.i. and Ncad.k.i.:p53 mammary glands whereas Ecad.k.o. and Ecad.k.o.:p53 were negative for p-Fgfr. Fgfr and GAPDH were used as loading controls. p-Fgfr, phospho-Fibroblast growth factor receptor; Ncadk.i., WAP::Cre;*Ecad^Ncad/fl^*; Ncadk.i.;p53, WAP::Cre;*Ecad^Ncad/fl^*;*p53^fl/+^*; Ecadk.o., WAP::Cre;*Ecad^fl/fl ^*; Ecadk.o.;p53, WAP::Cre;*Ecad^fl/fl^*;*p53^fl/+^*; Fgfr, Fibroblast growth factor receptor; GAPDH, Glyceraldehyde 3-phosphate dehydrogenase. Click here for file

Additional file 6 **Blocking of the Fgf pathway in mammary gland in-vitro organ culture induces down-regulation of downstream factors**. Ncad.k.i.:p53 mammary glands were incubated with either the Fgf inhibitor SU5402 or DMSO as a control for 24 hours at 37°C. Immunohistochemistry of tissue sections from these samples show a downregulation of p-Stat3 upon SU5402 exposure compared to control. Western blot analysis of protein lysates reveals the downregulation of p-Stat3, p-Erk and Snail1 after the treatment with SU5402 compared to control. GAPDH was used as a loading control. Ncadk.i.;p53, WAP::Cre;*Ecad^Ncad/fl^*;*p53^fl/+^*; Fgf, Fibroblast growth factor; DMSO, Dimethylsulfoxid; P-Stat3, phospho-Signal transducer and activator of transcription 3; p-Erk, phospho-extracellular signal-regulated kinase; GAPDH, Glyceraldehyde 3-phosphate dehydrogenase. Click here for file

Additional file 7 **Ecadk.o. and Ecadk.o.;p53 MG do not show any migration of alveolar epithelial cells. Ecadk.o., Ecadk.o.;p53 and control;p53 MG containing the ROSA26 allele were isolated at the third day of the tenth lactation cycle**. After whole mount X-gal staining the MG were sectioned and counterstained. Recombined alveolar epithelial cells do not migrate from the epithelial cell layer in the three samples depicted. Ecadk.o., WAP::Cre;*Ecad^fl/fl ^*; Ecadk.o.;p53, WAP::Cre;*Ecad^fl/fl^*;*p53^fl/+^*; control;p53, WAP::Cre; *Ecad^fl/+^*; *p53^fl/+^*; ROSA26, reverse orientation splice acceptor 26; X-gal, 5-bromo-4-chloro-indolyl-galactopyranoside. Click here for file
