# Supplementary figures and images for: Replacement of E-cadherin by N-cadherin in the mammary gland leads to fibrocystic changes and tumor formation
Source: Breast Cancer Res. 2011 Oct 26;13(5):R104. doi: 10.1186/bcr3046 (PMC3262217; doi:10.1186/bcr3046)

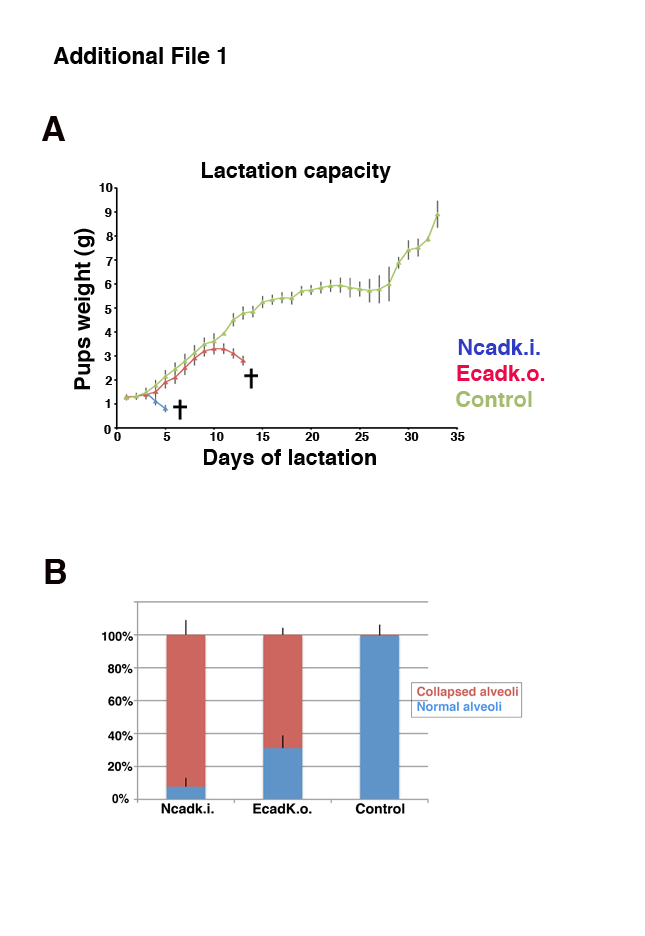

Supplement: Additional file 1 — Ncadk.i. and Ecadk.o. females show impaired lactation capacity and reduced number of intact alveoli. (A) To evaluate the function of the mammary gland, the weight of the pups of the corresponding females were monitored during the normal lactation period at the second lactation cycle. Control pups (green graph) gained weight progressively until reaching the time of weaning (approximately 20 days). Pups from Ecadk.o. females survived only 12 to 13 days (red graph) whereas pups from Ncadk.i. females died only two to three days after birth (blue graph). (B) The number of collapsed and intact alveoli was quantified in six individual H&E stained paraffin MG sections of Ncadk.i., Ecadk.o. and control mice from the third day of the second lactation cycle. The percentage of collapsed (red) or intact (blue) alveoli is depicted in relation to the entire amount of alveoli counted on the section (= 100%). Ncadk.i., WAP::Cre;EcadNcad/fl ; Ecadk.o., WAP::Cre;Ecadfl/fl ; Control, WAP::Cre;Ecadfl/+ [file bcr3046-S1.TIFF]

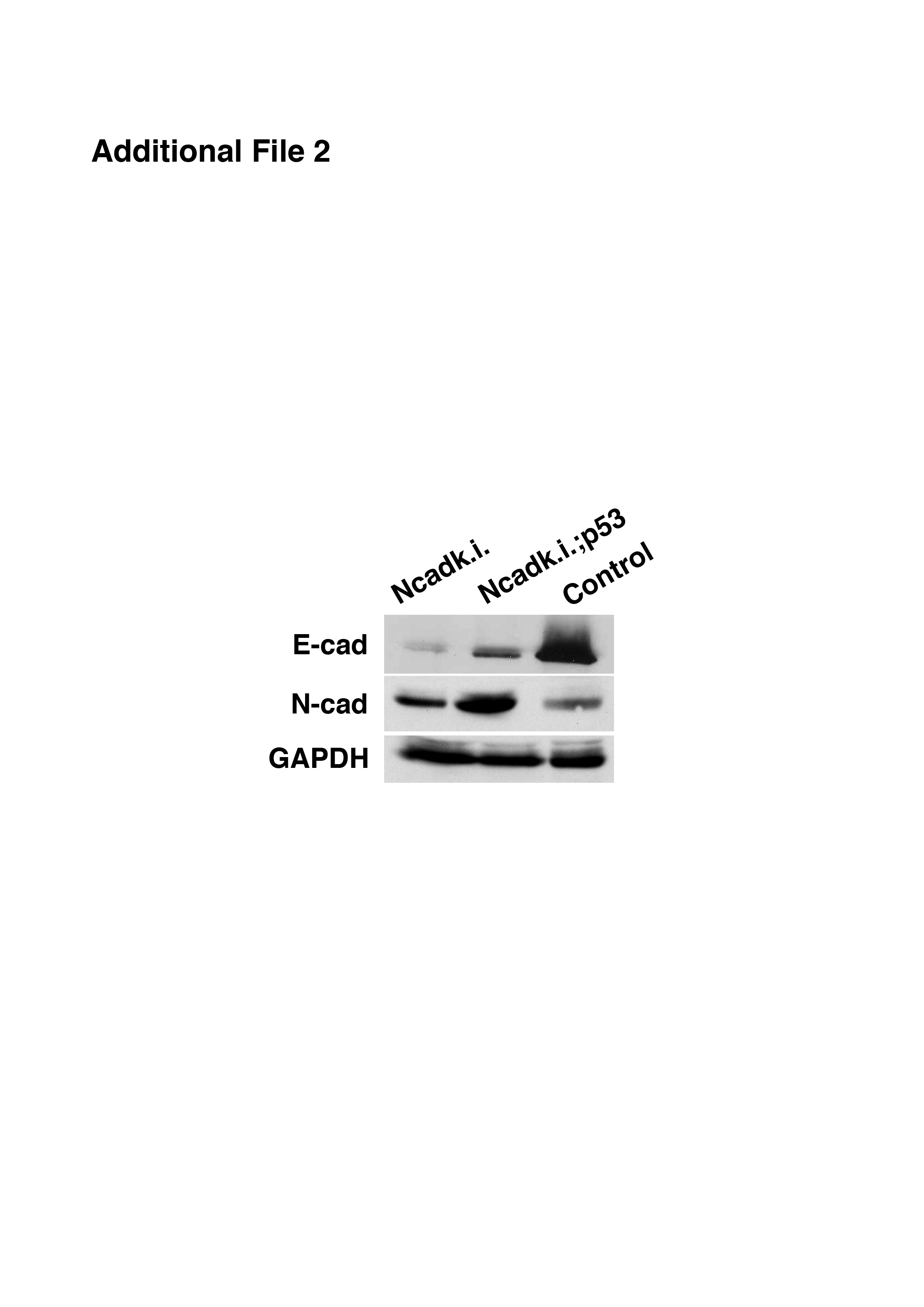

Supplement: Additional file 2 — Protein expression levels of Ecad and Ncad in Ncad.k.i. and Ncad.k.i.:p53 mammary glands. Western blot analysis of mammary gland protein lysates shows highly reduced protein levels of E-cad in both Ncad.k.i. and Ncad.k.i.:p53 animals as a result of efficient Cre mediated recombination. Ncad is expressed comparable to wildtype Ecad in the control sample. GAPDH was used as a loading control. E-cad, E-cadherin; Ncadk.i., WAP::Cre;EcadNcad/fl; Ncadk.i.;p53, WAP::Cre;EcadNcad/fl;p53fl/+; N-cad, N-cadherin; GAPDH, Glyceraldehyde 3-phosphate dehydrogenase. [file bcr3046-S2.TIFF]

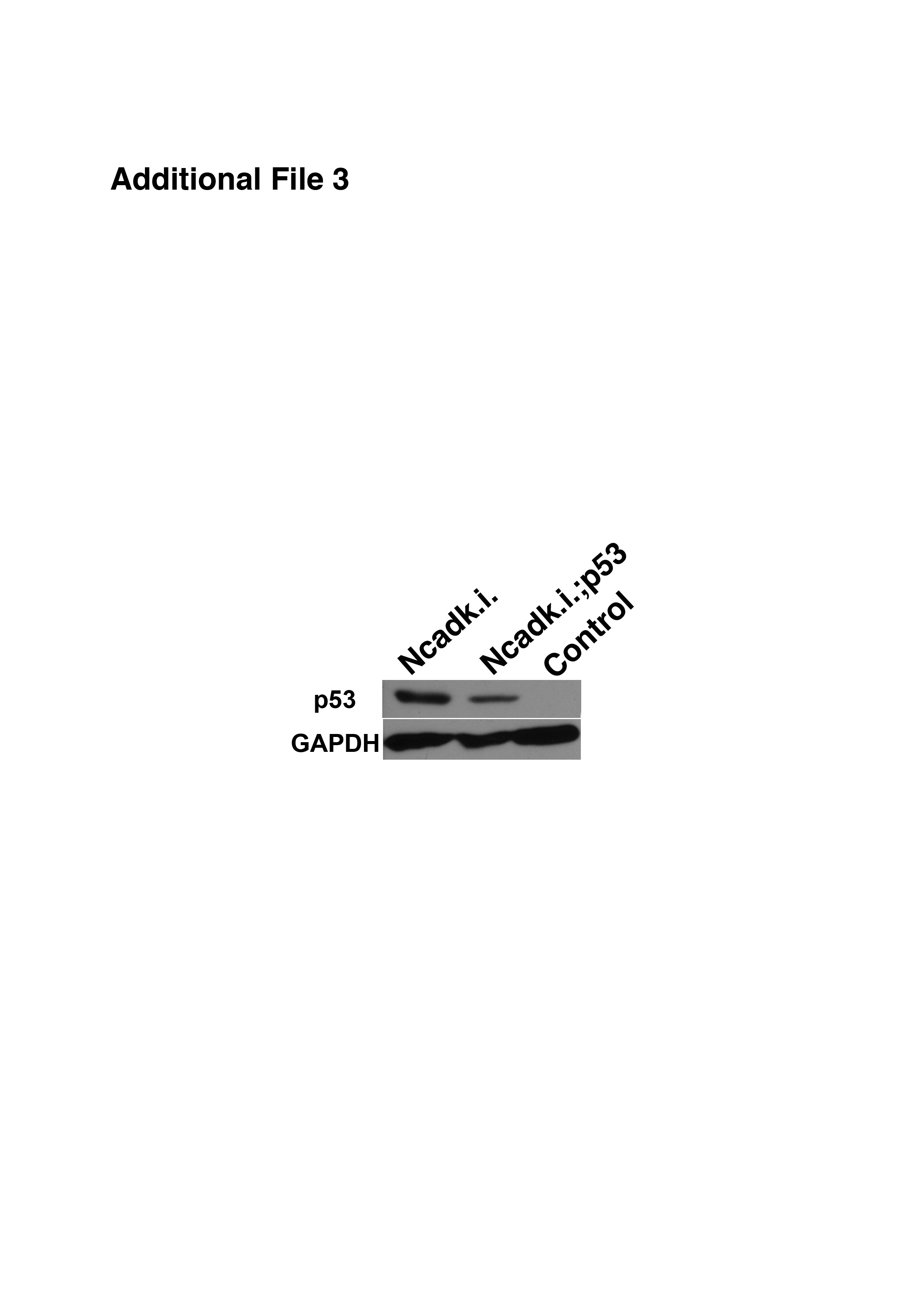

Supplement: Additional file 3 — Heterozygous p53 deletion leads to a marked decrease of active p53. Western blot analysis of mammary gland protein lysate reveals a approximate 50% decrease of p53 protein level in Ncad.k.i.:p53 compared to Ncad.k.i. GAPDH was used as a loading control. Ncadk.i., WAP::Cre;EcadNcad/fl; Ncadk.i.;p53, WAP::Cre;EcadNcad/fl;p53fl/+; p53, protein 53; GAPDH, Glyceraldehyde 3-phosphate dehydrogenase. [file bcr3046-S3.TIFF]

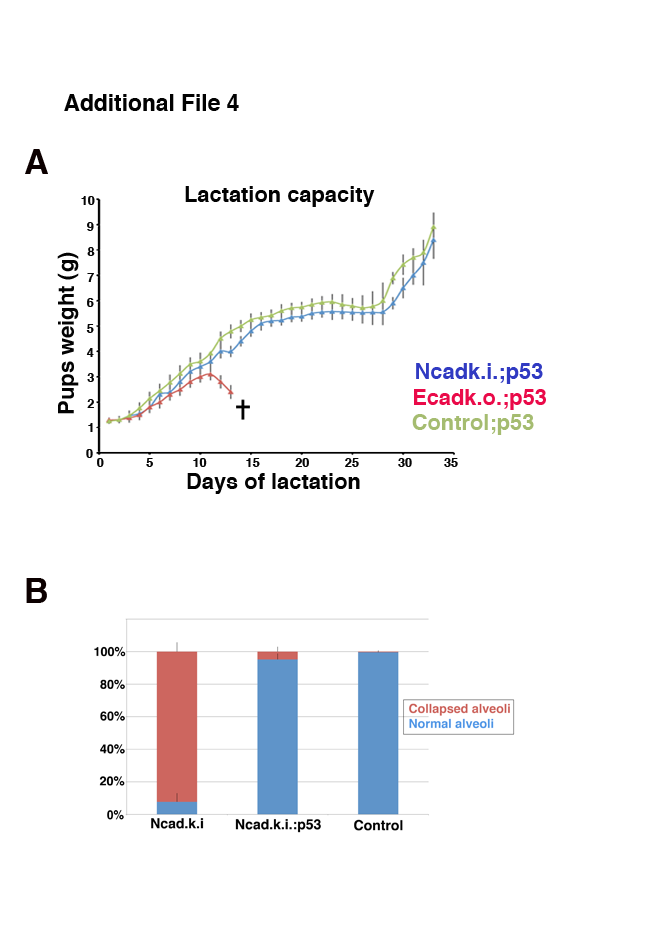

Supplement: Additional file 4 — The heterozygous deletion of p53 rescues the lactation capacity and the number of intact alveoli of Ncadk.i. but not of Ecadk.o. females. (A) The weights of pups from Ncadk.i.;p53 females (blue graph) monitored during the first four weeks after birth were comparable to the control (green), while the deletion of p53 in Ecadk.o. females had no effect on the survival rate of the offspring (red). (B) The number of collapsed and intact alveoli was quantified in six individual H&E stained paraffin MG sections of Ncadk.i., Ncadk.i.;p53 and control;p53 mice from the third day of the second lactation cycle. The percentage of collapsed (red) or intact (blue) alveoli is depicted in relation to the entire amount of alveoli counted on the section (= 100%). Ncadk.i.;p53, WAP::Cre;EcadNcad/fl;p53fl/+; control;p53, WAP::Cre; Ecadfl/+; p53fl/+; Ecadk.o.;p53, WAP::Cre;Ecadfl/fl;p53fl/+; Ncadk.i., WAP::Cre;EcadNcad/fl. [file bcr3046-S4.TIFF]

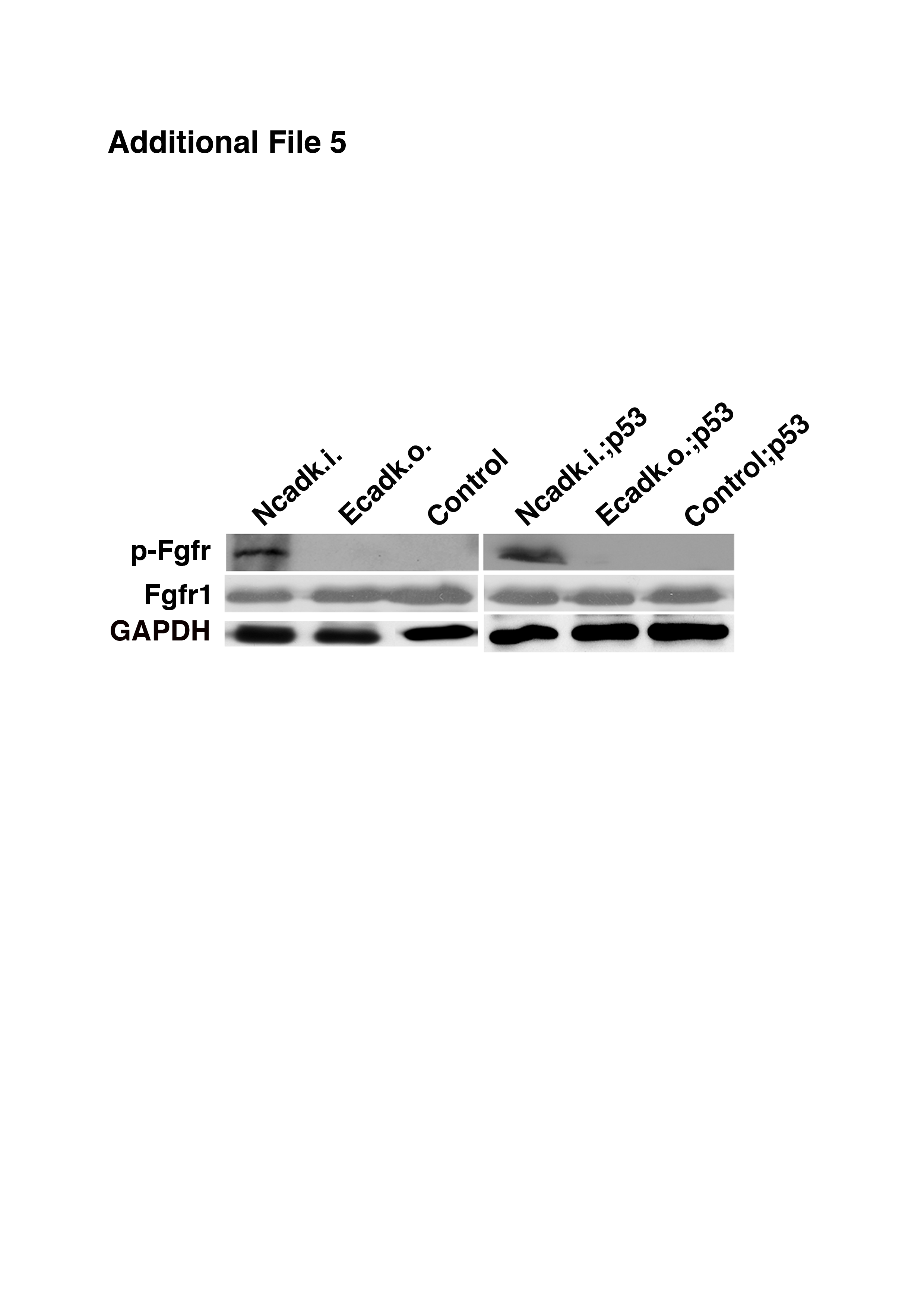

Supplement: Additional file 5 — Fgf receptor activation is detected in Ncad.k.i. and Ncad.k.i.:p53 but not in Ecad.k.o. nor Ecad.k.o.:p53 mammary glands. Western blot analysis of mammary gland protein lysates reveals the presence of p-Fgfr in both Ncad.k.i. and Ncad.k.i.:p53 mammary glands whereas Ecad.k.o. and Ecad.k.o.:p53 were negative for p-Fgfr. Fgfr and GAPDH were used as loading controls. p-Fgfr, phospho-Fibroblast growth factor receptor; Ncadk.i., WAP::Cre;EcadNcad/fl; Ncadk.i.;p53, WAP::Cre;EcadNcad/fl;p53fl/+; Ecadk.o., WAP::Cre;Ecadfl/fl ; Ecadk.o.;p53, WAP::Cre;Ecadfl/fl;p53fl/+; Fgfr, Fibroblast growth factor receptor; GAPDH, Glyceraldehyde 3-phosphate dehydrogenase. [file bcr3046-S5.TIFF]

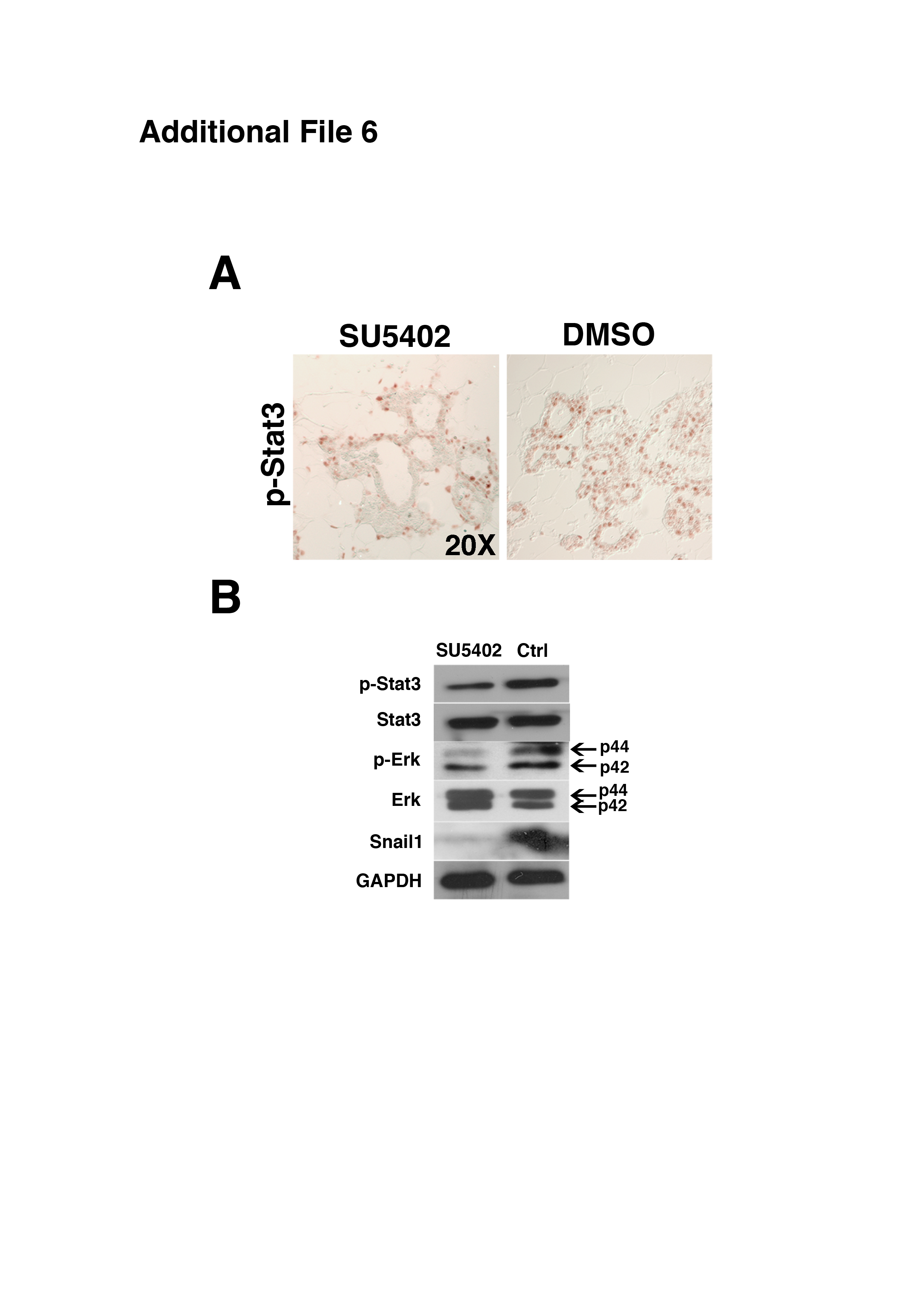

Supplement: Additional file 6 — Blocking of the Fgf pathway in mammary gland in-vitro organ culture induces down-regulation of downstream factors. Ncad.k.i.:p53 mammary glands were incubated with either the Fgf inhibitor SU5402 or DMSO as a control for 24 hours at 37°C. Immunohistochemistry of tissue sections from these samples show a downregulation of p-Stat3 upon SU5402 exposure compared to control. Western blot analysis of protein lysates reveals the downregulation of p-Stat3, p-Erk and Snail1 after the treatment with SU5402 compared to control. GAPDH was used as a loading control. Ncadk.i.;p53, WAP::Cre;EcadNcad/fl;p53fl/+; Fgf, Fibroblast growth factor; DMSO, Dimethylsulfoxid; P-Stat3, phospho-Signal transducer and activator of transcription 3; p-Erk, phospho-extracellular signal-regulated kinase; GAPDH, Glyceraldehyde 3-phosphate dehydrogenase. [file bcr3046-S6.TIFF]

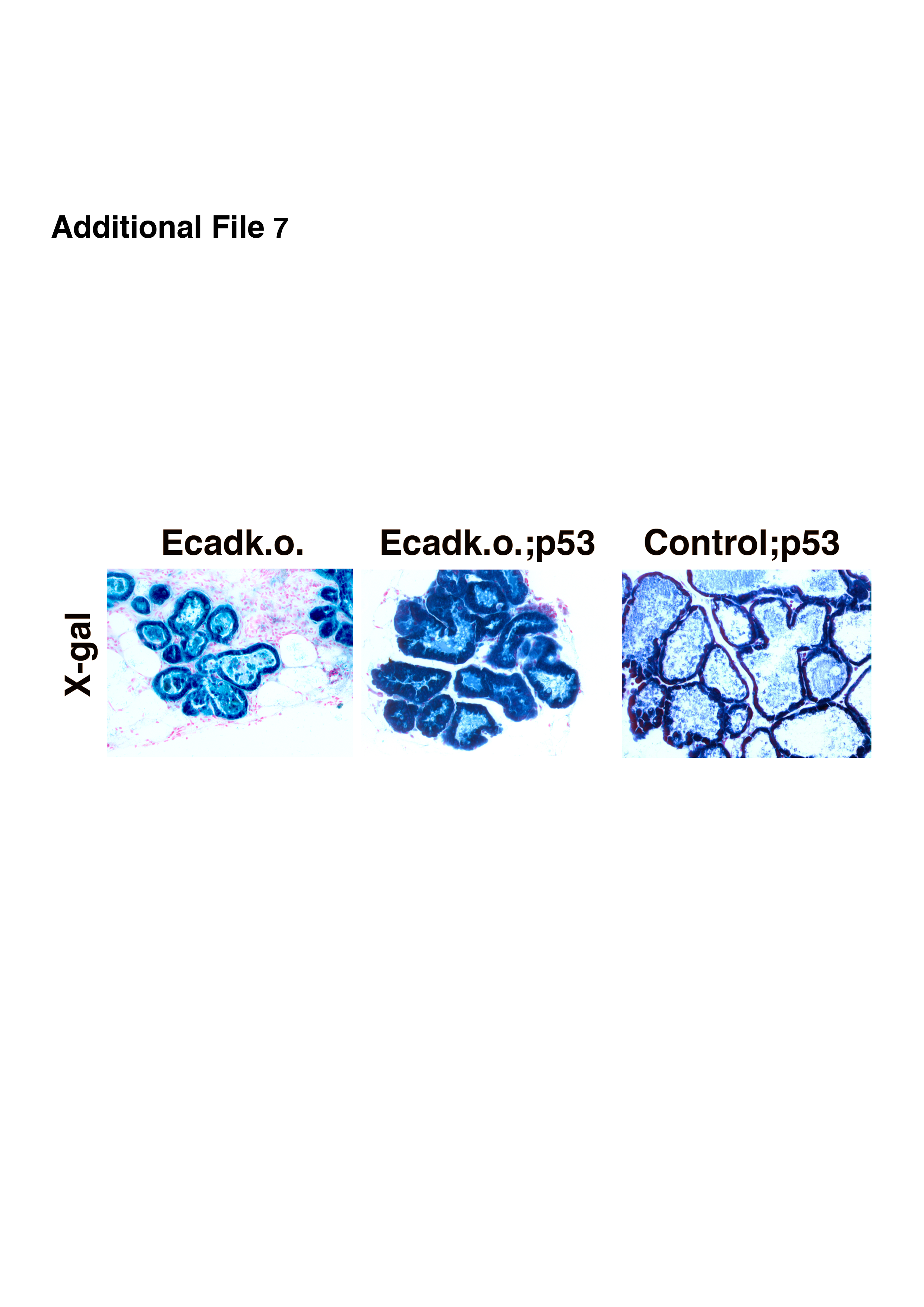

Supplement: Additional file 7 — Ecadk.o. and Ecadk.o.;p53 MG do not show any migration of alveolar epithelial cells. Ecadk.o., Ecadk.o.;p53 and control;p53 MG containing the ROSA26 allele were isolated at the third day of the tenth lactation cycle. After whole mount X-gal staining the MG were sectioned and counterstained. Recombined alveolar epithelial cells do not migrate from the epithelial cell layer in the three samples depicted. Ecadk.o., WAP::Cre;Ecadfl/fl ; Ecadk.o.;p53, WAP::Cre;Ecadfl/fl;p53fl/+; control;p53, WAP::Cre; Ecadfl/+; p53fl/+; ROSA26, reverse orientation splice acceptor 26; X-gal, 5-bromo-4-chloro-indolyl-galactopyranoside. [file bcr3046-S7.TIFF]
